# Pre-frontal Cortical Activity During Walking and Turning Is Reliable and Differentiates Across Young, Older Adults and People With Parkinson's Disease

**DOI:** 10.3389/fneur.2019.00536

**Published:** 2019-05-22

**Authors:** Samuel Stuart, Valeria Belluscio, Joseph F. Quinn, Martina Mancini

**Affiliations:** ^1^Department of Neurology, Oregon Health and Science University, Portland, OR, United States; ^2^Department of Movement, Human and Health Sciences, Università degli Studi di Roma Foro Italico, Rome, Italy

**Keywords:** Parkinson's disease, fNIRS, turning, walking, pre-frontal cortex

## Abstract

**Introduction:** Mobility declines with age and further with neurodegenerative disorders, such as Parkinson's disease (PD). Walking and turning ability, in particular, are vital aspects of mobility that deteriorate with age and are further impaired in PD. Such deficits have been linked with reduction in automatic control of movement and the need for compensatory cognitive cortical control via the pre-frontal cortex (PFC), however the underlying neural mechanisms remain unclear. Establishing and using a robust methodology to examine PFC activity during continuous walking and turning via mobile functional near infra-red spectroscopy (fNIRS) may aid in the understanding of mobility deficits and help with development of appropriate therapeutics.

This study aimed to: (1) examine test re-test reliability of PFC activity during continuous turning and walking via fNIRS measurement; and (2) compare PFC activity during continuous turning and walking in young, old and Parkinson's subjects.

**Methods:** Twenty-five young (32.3 ± 7.5 years), nineteen older (65.4 ± 7.0 years), and twenty-four PD (69.3 ± 4.1 years) participants performed continuous walking and 360° turning-in-place tasks, each lasting 2 min. Young participants repeated the tasks a second time to allow fNIRS measurement reliability assessment. The primary outcome was PFC activity, assessed via measuring changes in oxygenated hemoglobin (HbO_2_) concentrations.

**Results:** PFC activity during continuous walking and turning was moderately reproducible (Intra-class correlation coefficient = 0.67). The PD group had higher PFC activation than young and older adults during walking and turning, with significant group differences for bilateral PFC activation (*p* = 0.025), left PFC activation (*p* = 0.012), and the early period (first 40 s) of walking (*p* = 0.007), with greater activation required in PD. Interestingly, older adults had similar PFC activation to young adults across conditions, however older adults required greater activation than young adults during continuous turning, specifically the early period of the turning task (Cohens *d* = 0.86).

**Conclusions:** PFC activity can be measured during continuous walking and turning tasks with acceptable reliability, and can differentiate young, older and PD groups. PFC activation was significantly greater in PD compared to young and older adults during walking, particularly when beginning to walk.

## Introduction

Decline in mobility occurs with age (1–3), and gait and turning impairments, which are central to reduction in independence, with links to increased falls risk (4–6), occur early in Parkinson's disease (PD) (7–9). Previous studies have shown that gait and turning are slower in PD, with shorter steps during walking and a greater number of steps required during turns (10–12). Turns during walking have been found to be impaired in PD (13, 14), and continuous 360° turning-in-place is particularly complex for people with PD and elicits intermittent mobility issues (15), such as freezing of gait (11, 16). While the motor contributions of mobility decline in older adults and people with PD have been well-studied through static imaging assessments (1), considerable evidence is building for non-motor contributions, such as the role of cognition (17, 18). Deficits in cognition can occur with age and are common in PD, with early impairment of executive function, visuo-spatial ability, working memory, and attention (19). Executive-attention may be an important contributor to gait and turning control in older adults and more so in PD, with associative and dual-task studies highlighting a strong association between them (20–25). Executive-attentional projections stem from the pre-frontal cortex (PFC), and may become over-active during gait or turning in PD compared to healthy controls (26, 27) to compensate for the impaired basal ganglia output that affects the automaticity of movement (28).

Technological advancement has recently allowed monitoring of PFC activity during mobility tasks, using methods such as mobile functional near infrared spectroscopy (fNIRS) (29) or electroencephalography (EEG) head caps. These devices are a valid means to measure cortical activity in humans (30, 31) and can be used in a variety of different motor tasks, from static seated tasks to more dynamic mobility tasks, such as walking in different conditions (32–41). The majority of previous studies that have used mobile fNIRS or EEG during walking have examined healthy young or older adults (33), with few investigating changes in PD (32, 36–40). Measurement of PFC activity via fNIRS in individuals with PD may be particularly useful, as it is relatively quick and easy to set-up and use compared to EEG (42). The previous studies that have examined PFC response using fNIRS in PD during dynamic mobility tasks have generally shown increased PFC activity in people with PD, generally tested ON dopaminergic medication, compared to older adults (32, 33, 37–41, 43). However, the majority of these previous studies have used different protocols and two recent reviews of this research area have highlighted methodological issues with previous studies that may prevent the generalization of results (32, 33). In fact, the majority of the studies have examined relatively small cohorts (*N* < 15), and have lacked short-separation reference channels (emitter-detector distances <1.5 cm apart) that are used remove peripheral hemodynamic response (i.e., increased skin or superficial blood flow due to physical activity rather than cortical activity) from regular channels (3 cm apart) (44), which limits or possibly inflates interpretation of results. Similarly, previous studies have provided no information on the reliability of findings, with little information on how reliable findings are in different populations, such as differences with age or neurological disease. Therefore, using a mobile fNIRS system with short-separation reference channels, this study aimed to: (1) examine test re-test reliability of PFC activity during turning and walking tasks in young adults; and (2) compare PFC activity during turning and walking tasks in young, old and people with PD.

## Methods

### Participants

A total of 68 participants participated in this study; twenty-five young adults (YA), nineteen older adults (OA) and twenty-four people with PD. Young and older adult participants were recruited via local advertisement (i.e., posters within campus notice boards and e-mails circulated to students and staff members). People with PD were recruited from local Neurology clinics via referrals from movement disorder specialist neurologists. All study procedures were approved by the Oregon Health and Science University Institutional Review Board, with written informed consent obtained before participation.

Participants were included if they were aged 20–40 (young adults) or 50–90 (older adults or PD) years, able to stand or walk for 2 min without assistance. People with PD were included if they had a diagnosis of PD as defined by the UK Brain Bank criteria, Hoehn and Yahr score II-IV and were taking anti-Parkinsonian medication. Exclusion criteria were; musculoskeletal, vestibular, visual, or other medical condition that affected gait or balance.

### Experimental Design and Equipment

Participant characteristics of age, sex, height, and weight were recorded. Global cognition was measured with the Montreal Cognitive Assessment (MoCA) (45). All testing in people with PD was performed in the OFF medication state, ~12 h after taking last medication dosage. Disease severity was measured using the Movement Disorders Society (MDS-revised) unified Parkinson's disease rating scale (MDS-UPDRS) (46), freezing-status was measured using the new Freezing of Gait Questionnaire (nFOGQ) (47), and levodopa equivalent daily dosage (LEDD) was calculated (48). Specifically, out of twenty-four participants with PD, twelve had freezing of gait, as reported by the nFOGQ (nFOGQ>1).

The participants completed two different motor tasks at self-selected pace; (1) a 2-min 360° turning-in-place task, alternating 360° turning to the left and 360° turning to the right; and (2) a 2-min walking task. Each condition included a baseline of 20 s of standing at the start and end of the task (with 80 s of performing the turning or walking task in between). The walking condition was conducted over-ground with participants walking back and forth over a 9 m straight path, with a 180° degree turn at each end. Condition order was randomized for the participants, with breaks between tasks if needed. A research assistant walked with the participants and stood by the participants during turning to ensure their safety.

Test re-test reliability of the fNIRS measurement of PFC activity during the continuous turning and walking trials was conducted within the young adult subjects. The young adult group performed the same walking and turning tasks for a second time after having the fNIRS device removed and replaced following a short break (~5–10 min).

A mobile fNIRS system (Oxymon, Artinis, Netherlands) was used to record changes in oxygenated hemoglobin (HbO_2_) and deoxygenated hemoglobin (HHb) within the PFC at a sampling rate of 50 Hz. Distance from transmitter to detector was 3.5 cm (38) and data was collected and processed in line with previous studies (32, 33). Additionally, two short-separation reference channels at a distance of 1.5 cm (one left and one right hemisphere) were used to allow for removal of peripheral interference (i.e., from blood flow changes in the extra-cerebral layers of the head) in the long source-detector separation channels (44).

### Data Processing and Analysis

All processing of fNIRS signals followed current recommendations where possible. A digitizer (PATRIOT, Polhemus, VT, USA) was used to provide 3-dimensional morphological locations for cortical regions of interest relative to scalp position and the fNIRS optode measure. Data from the digitizer was entered into the software package NIRS-statistical package metric mapping (NIRS-SPM, http://www.nitrc.org/projects/nirs_spm) (49), which was implemented within MATLAB 2017a (Mathworks, MA, USA). NIRS-SPM allows registration of fNIRS channel data onto the Montreal Neurological Institute (MNI) standard brain space (50). NIRS-SPM used probabilistic registration of the fNIRS co-ordinate data to determine channels that related to ROIs at the group level [described in detail elsewhere (51)]. HbO_2_ changes were recorded bilaterally (left and right) within the pre-frontal cortex (PFC). The Brodmann areas (BA) that corresponded to the PFC consisted of BA9 and BA10 for all of the participants.

The fNIRS data were processed within custom-made MATLAB algorithms, which consisted of several steps:

1. **Data filtering**: After zeroing data to the initial time-point, a low-pass filter with a cut-off frequency of 0.14 Hz, based on canonical hemodynamic response function, removed high-frequency noise (52).
2. **Baseline correction:** Removing the median of the initial 20 s of baseline standing fNIRS signal from the entire trial (i.e., subtracting the baseline period from the rest of the signal).
3. **Reference channel correction:** This step corrected signal distortions due to artifact caused by breathing, cardiac cycle, vasomotor or other error related to movement (53, 54). First, a scaling factor was determined by detecting the peaks (positive and negative) of the heart rate within the long and short-separation channel signals, then dividing them to produce the scaling factor. This was then used to remove the noise detected within the short-separation reference channels within the long-separation channels. The following formula describe the reference channel correction:
  $$\begin{array}{l} Scaling\;factor=\frac{Peak\;to\;peak\;difference\;in\;heart\;rate\;in\;long\;seperation\;channel}{\;Peak\;to\;peak\;difference\;in\;heart\;rate\;in\;short\;separation\;channel}\\ fNIRS\;signal\;=long\;separation\;channel\;signal\\ \;-\left(short\;separation\;channel\;signal\;\times Scaling\;factor\right) \end{array}$$
4. **Visual signal inspection:** All of the fNIRS signals were visually examined to ensure divergence between the HbO_2_ and HHb traces. This step allowed exclusion of trials that had poor fNIRS signal collection from participants, as a lack of divergence in HbO_2_ and HHb indicates noise interference.
5. **Averaging across fNIRS channels:** In line with previous research (32), bilateral signals from fNIRS optodes over the PFC were median averaged for further analysis. We also median averaged the left and right sided fNIRS optodes separately for further analysis.

### Primary Outcome

The primary outcome for this study was change in oxygenated hemoglobin (HbO_2_) from baseline standing to walking or turning, which is a proxy for cortical activation. The fNIRS system used emitter-detector pairs to emit light into the skull that diffused through brain tissues resulting in scattering of multiple photons (55). These photons were then detected by the fNIRS detector channels when exiting the skull after passing through the cortical brain layers (typically ~1–2 cm deep, with an emitter-detector optode distance of 3.5 cm). Importantly, HbO_2_, and HHb have different absorption rates for different wavelengths of near-infrared light, which can be analyzed with Beer-Lamberts law equations (56) within the fNIRS software to calculate the relationship between an exciting photon intensity and incident photon intensity to derive changes in HbO_2_ and HHb (57). Therefore, the fNIRS system measured optical density of the raw signal and converted this to HbO_2_ and HHb using Beer-Lamberts law (57). HbO_2_ rather than HHb was reported as our primary outcome due to its sensitivity to walking and cognitive tasks (58, 59). Additionally, changes in HbO_2_ concentration within local brain capillary networks are caused by neuron firings with brain activity, which is commonly referred to as neurovascular coupling (60). Relative changes from baseline standing (initial 20 s) in HbO_2_ concentration was reported in an attempt to account for between individual physiological variations (33); see below calculations.

Walking and turning periods;

1. Early = Median HbO_2_ first 40 s of task—Median HbO_2_ initial standing period (20 s)
2. Late = Median HbO_2_ second 40 s of task—Median HbO_2_ initial standing period (20 s)

### Statistical Analysis

Statistical analysis was conducted in SPSS (v.24, IBM, Armonk, NY, USA) and Shapiro-Wilks tests determined data normality with parametric analysis used throughout. Mean differences with paired-sample *t*-tests, intra-class correlation coefficients (Absolute Agreement: ICC_2, 1_) and Bland-Altman plots with 95% limits of agreement (LoA 95%) were used to determine the test re-test reliability of PFC activity measurement via fNIRS between the first and second data captures in young adults. Acceptance ratings for ICCs were set at excellent (>0.75), moderate (0.40–0.75), and poor (<0.40) agreement (61).

PFC HbO_2_ activation during turning and walking in young, older and PD groups was reported via mean and standard deviation. Separate linear mixed-effect models compared groups (YA, OA, PD), Periods (Early vs. Late) and PFC regions (Left vs. Right), with a random intercept for each subject within the models. *Post-hoc* independent *t*-tests explored significant differences between specific groups (YA vs. OA, YA vs. PD, OA vs. PD). We also compared between groups differences using Cohen's *d* effect sizes based on mean (SD) scores. Effect sizes were interpreted as small (0.2), medium (0.5), and large (0.8) (61). Due to the exploratory nature of the analysis, statistical significance was set at *p* < 0.05.

## Results

### Participants

Demographic characteristics of the participants are shown in Table 1. Groups were significantly different for age (*p* < 0.001), but age was not significantly different between older adults and PD groups (*p* = 0.166). Similarly, young adults tended to be taller than the older adults and PD subjects, but the older adults and PD groups did not significantly differ for height (*p* = 0.146). The groups were well-matched for gender (*p* = 0.160), weight (*p* = 0.287), and cognitive ability (*p* = 0.227).

**Table 1:** Participant demographic characteristics.

	**YA (*n* = 25) Mean (SD)**	**OA (*n* = 19) Mean (SD)**	**PD (*n* = 24) Mean (*SD*)**	**Group *p***
**DEMOGRAPHICS**				
Age (years)	32.3 ± 7.5	65.38 (6.95)	69.29 (4.05)	<0.001^*^
Gender	F (15)/M (10)	F (10)/M (9)	F (8)/M (16)	0.160
Height (cm)	1.70 (0.11)	1.67 (0.10)	1.62 (0.13)	0.035^*^
Weight (kg)	70.86 (13.95)	73.51 (15.42)	77.47 (14.56)	0.287
MoCA	28.67 (1.24)	27.47 (3.91)	27.69 (3.29)	0.227
**CLINICAL**				
Disease duration (years)	–	–	9.88 (6.37)	–
MDS-UPDRS III	–	–	36.54 (11.90)	–
FOGQ	–	–	6.54 (8.18)	–
LEDD	–	–	861.34 (499.07)	–
H&Y	–	–	I (0)/II (21)/III (3)	–

*UPDRS III, unified Parkinson's disease rating scale—motor subsection, FOGQ, freezing of gait questionnaire, LEDD, levodopa equivalent daily dosage, H&Y, Hoehn and Yahr scale*.

* *Significance p < 0.05 should be in bold*.

### Test Re-test Reliability of fNIRS Measurement During Turning and Walking

Following removal of fNIRS data that had poor signal quality, data from 19 young adult subjects were analyzed for walking (*n* = 6 young adult walking trials were excluded following visual signal inspection: 4 first trial and 2 second trial errors) and 25 subjects were analyzed for turning to determine test re-test reliability of fNIRS measurement.

**Figure 1:**
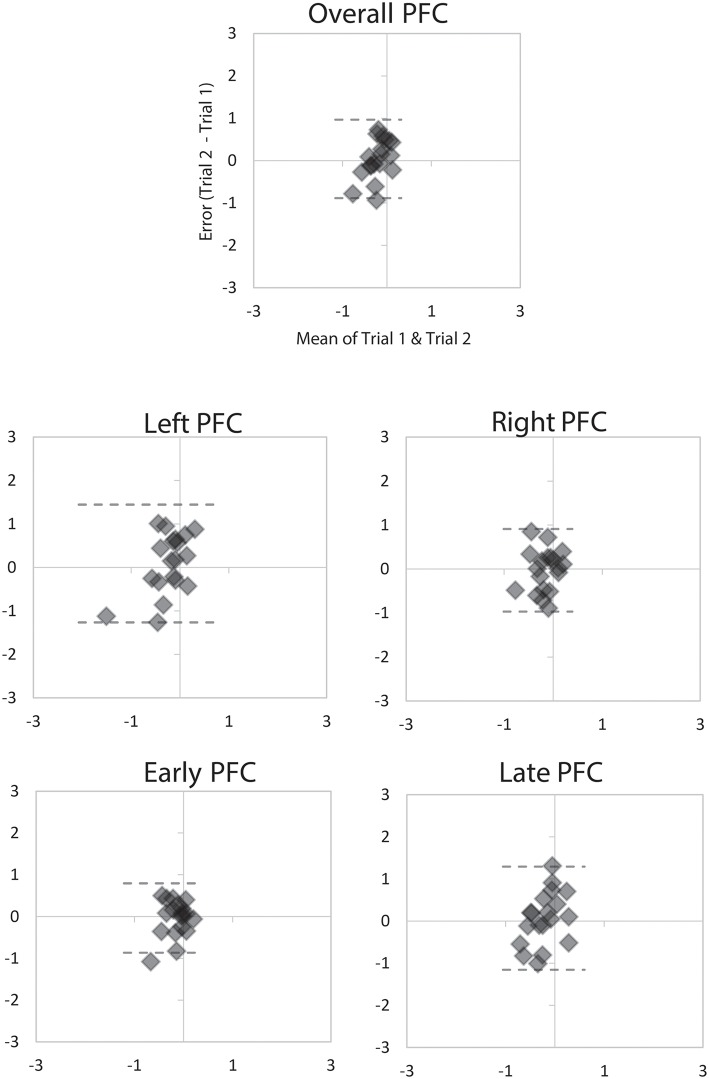
Bland Altman plots demonstrating agreement between the two 2-min trials of walking for overall PFC activity, Left and Right PFC activity, and Early and Late PFC activity. Dashed lines represent LoA.

**Figure 2:**
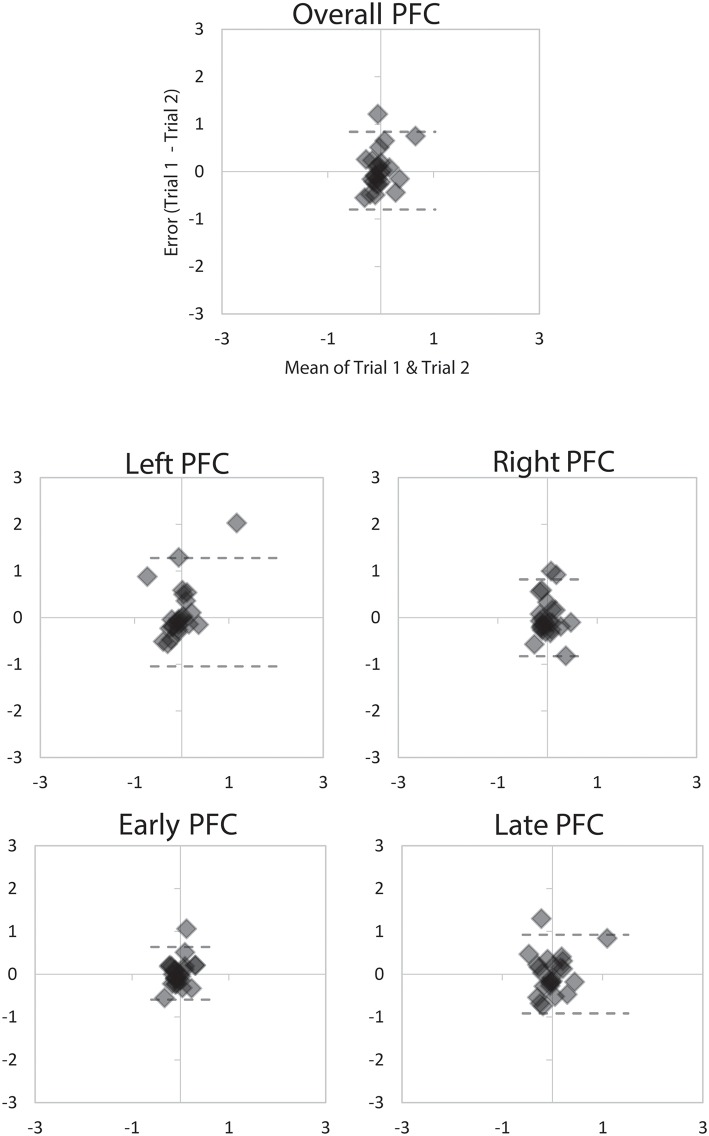
Bland Altman plots demonstrating agreement between the two 2-min trials of turning for overall PFC activity, Left and Right PFC activity, and Early and Late PFC activity. Dashed lines represent LoA.

On average, there was moderate (ICC_2, 1_ = 0.67) reliability of PFC activity measured via mobile fNIRS during turning and walking in young adults (Table 2). There were no significant differences between any of the outcomes over the two sessions, with very low average difference between testing sessions (Mean Difference = 0.03 μm, Figures 1, 2). Reliability across the different time periods (early vs. late) and regions of interest (Left, Right or combined) within the PFC ranged from moderate (ICC_2, 1_ = 0.52) to excellent (ICC_2, 1_ = 0.79). Importantly, the primary outcome over the duration of the motor tasks (combined left and right PFC regions) had moderate reliability (turning; ICC_2, 1_ = 0.67, walking; ICC_2, 1_ 0.71) (Table 2).

**Table 2:** Reliability of fNIRS recording of PFC activity in young adults.

**Task**	**Time**	**PFC region**	**Trial 1 Mean (SD)**	**Trial 2 Mean (SD)**	**Mean Difference^a^**	***p***	**ICC_**2, 1**_ (95% CI)**	**LoA 95%**
Turning (*n* = 25)	Overall	B/L	−0.02 (0.25)	−0.01 (0.33)	0.02	0.859	0.673 (0.245 to 0.857)	0.819
		L	−0.02 (0.19)	0.03 (0.55)	0.12	0.297	0.512 (−0.093 to 0.784)	1.162
		R	0.03 (0.28)	0.01 (0.27)	−0.01	0.745	0.658 (0.213 to 0.850)	0.823
	Early	B/L	−0.02 (0.19)	−0.02 (0.27)	0.02	0.863	0.793 (0.517 to 0.911)	0.616
	Late	B/L	0.01 (0.33)	0.01 (0.43)	0.00	0.959	0.709 (0.316 to 0.875)	0.919
Walking (*n* = 19)	Overall	B/L	−0.19 (0.24)	−0.18 (0.41)	0.04	0.940	0.714 (0.239 to 0.891)	0.925
		L	−0.23 (0.33)	−0.19 (0.64)	0.09	0.786	0.685 (0.164 to 0.880)	1.355
		R	−0.15 (0.32)	0.18 (0.36)	−0.03	0.682	0.709 (0.231 to 0.889)	0.938
	Early	B/L	−0.16 (0.26)	−0.17 (0.34)	−0.04	0.897	0.520 (−0.511 to 0.846)	0.831
	Late	B/L	−0.17 (0.34)	−0.17 (0.51)	0.07	0.977	0.707 (0.220 to 0.888)	1.224
Average	Overall	B/L	–	–	0.03	–	0.668 (0.154 to 0.867)	0.961

a Trial 2 minus Trial 1, PFC, pre-frontal cortex; ICC, Intra-class correlation coefficient; CI, confidence interval; LoA, limits of agreement; B/L, bilateral; L, left; R, right. * p < 0.05.

### PFC Activity During Continuous Turning and Walking in Young, Old, and Parkinson's

Table 3 shows the relative change in HbO_2_ during continuous turning and walking in the groups. Overall, the PD group had higher average levels of PFC activation during turning and walking than young or older adults across the majority of the time-points and PFC regions. Older adults also had higher average PFC activation during turning and walking compared to young adults.

**Table 3:** Relative change in HbO_2_ during turning and walking in young, old and Parkinson's participants.

**Task**	**Time**	**PFC region**	**YA Mean (SD)**	**OA Mean (SD)**	**PD Mean (SD)**	**Group *p***	**Period (early vs. late) *p***	**Hemisphere (L vs. R) *p***	***Post-hoc***
Turning	Overall	B/L	−0.01 (0.25)	0.01 (0.30)	0.06 (0.35)	0.464	0.647	0.059	–
		L	−0.06 (0.34)	0.03 (0.30)	0.05 (0.28)	0.198			–
		R	0.03 (0.28)	0.08 (0.33)	0.09 (0.41)	0.573			–
	Early	B/L	−0.17 (0.19)	0.04 (0.31)	0.10 (0.26)	0.114			–
	Late	B/L	−0.01 (0.31)	0.02 (0.41)	0.05 (0.46)	0.646			–
Walking	Overall	B/L	−0.26 (0.28)	−0.25 (0.36)	−0.03 (0.37)	0.025^*^	0.012^*^	0.185	0.025^*^ **(YA** **<** **PD)**
		L	−0.29 (0.32)	−0.21 (0.33)	−0.03 (0.37)	0.012^*^			0.017^*^ **(YA** **<** **PD)**
		R	−0.21 (0.36)	−0.18 (0.40)	0.02 (0.49)	0.065			–
	Early	B/L	−0.18 (0.30)	−0.14 (0.32)	0.07 (0.29)	0.007^*^			0.008^*^ **(YA** **<** **PD)** 0.037^*^(OA **<** **PD)**
	Late	B/L	−0.29 (0.37)	−0.25 (0.43)	−0.10 (0.44)	0.136			–

* *Significance p < 0.05 should be in bold. PFC, pre-frontal cortex; YA, young adults; OA, older adults; PD, Parkinson's disease; B/L, bilateral; L, left; R, right*.

During walking the groups were significantly different for overall PFC activation (*p* = 0.025), left PFC activation (*p* = 0.012) and for the early period (first 40 s) of walking (*p* = 0.007) (Table 3). There was also a significant difference in PFC activation between early and late periods of walking across the groups (*p* = 0.012), with higher PFC activity in the early period. *Post-hoc* testing indicated that the PD group had significantly higher PFC activation during the early period of walking compared to the young (*p* = 0.008) and older adults (*p* = 0.037). The PD group also had significantly higher overall (*p* = 0.025) and left side (*p* = 0.017) PFC activation than the young adults. However, the older adult group did not have significantly different PFC activation compared to the young adults, with small effect size differences between groups (Table 4). Overall, the PD group had moderate to large differences (Cohen's *d* 0.52 to 0.86) in PFC activation during walking compared to young and older adults (Table 4), particularly for the early period and left PFC region.

**Table 4:** Effect sizes (Cohens d) for differences in HbO_2_ during turning and walking between groups.

**Task**	**Time**	**PFC region**	**YA vs. OA**	**YA vs. PD**	**OA vs. PD**
Turning	Overall	B/L	0.08	0.24	0.16
		L	0.28	0.36	0.07
		R	0.17	0.18	0.03
	Early	B/L	**0.86**	**1.21**	0.22
	Late	B/L	0.09	0.16	0.07
Walking	Overall	B/L	0.03	**0.72**	**0.62**
		L	0.25	**0.77**	**0.52**
		R	0.08	**0.55**	0.45
	Early	B/L	0.13	**0.86**	**0.71**
	Late	B/L	0.10	0.48	0.35

*Bold indicates effect size > 0.50 (medium effect). PFC, pre-frontal cortex; YA, young adults; OA, older adults; PD, Parkinson's disease; B/L, bilateral; L, left; R, right*.

Tables 3, 4 demonstrate that the effect of turning on PFC activation was similar across groups, as during continuous 360° turning there were no significant group differences for any outcome. However, there was a trending difference between PFC regions during turning (left vs. right, *p* = 0.059), with higher right PFC activity compared to left (Table 3). Despite the lack of a significant difference, effect sizes showed that the early period of turning differentiated the young group from the older adult and PD groups (Table 4; Cohens *d* of 0.86 and 1.21, respectively), with young adults having the lowest activation of the three groups (Table 3).

## Discussion

To the best of our knowledge, this is the first study to examine the test re-test reliability of PFC activity measurement via fNIRS during continuous walking and turning tasks. Findings demonstrate that the measurement of PFC activity during continuous (2 min) turning or walking has acceptable reliability, as there was little difference in HbO_2_ measurement between separate re-test trials. With a growing interest in understanding brain activity during motor tasks, our results contribute to the development of robust protocols to examine PFC activity using fNIRS during continuous walking or turning tasks.

This study also reports differences in PFC activation during continuous (2 min) 360° turning and walking in young adults, older adults and people with PD. Specifically, people with PD had significantly higher PFC activation during walking compared to young and older adults; however, older adults were not significantly different compared to young adults. Increased PFC activation may indicate greater cognitive demand during walking in PD due to impaired movement automaticity.

### Reliability of fNIRS Monitoring of PFC Activation During Turning and Walking

The mobile fNIRS device used in this study is a commercial device that allows access to the raw data that registers HbO_2_ concentration and subsequent implementation of our custom-made algorithms for data analysis. Re-test reliability of HbO_2_ signal recorded with the mobile fNIRS device was conducted within our young adult group with some variations across the conditions (walking or turning), as well as when the HbO_2_ signal was broken into different PFC hemispheres (left or right) or trial times (early or late). When using our fNIRS system with short-separation reference channels and fixed data analysis pipeline we found moderate reliability (ICC_2, 1_ 0.67) of the PFC HbO_2_ signal during the continuous walking and turning tasks. These results, together with small LoA between trials, indicated that we could be confident that this signal is reliable, as reported in Figures 1, 2. However, there were changes in reliability when breaking the fNIRS signal down into individual hemispheres or times of the signal (early vs. late), which is similar to previous static fNIRS findings (62). On the basis of our results, it can be stated that HbO_2_ outcomes measured using a mobile fNIRS device during continuous (2 min) walking or turning are relatively stable. However, when breaking the signal into specific features, such as hemispheres or time-periods, the stability of the HbO_2_ signal can be altered.

### Impact of Aging and PD on PFC Activation During Turning and Walking

This study confirms that PFC activity, measured through mobile fNIRS, can show differences in PFC activation during continuous walking and turning between young, older and subjects with PD. Specifically, we found that people with PD, OFF their medication, had significantly higher PFC activation during walking than young and older adults, in line with previous studies where PD subjects were ON medication (32, 33, 37–40). Higher levels of PFC activation during continuous walking, particularly the early period of walking, in PD compared to the other groups likely reflects the need to use executive-attentional resources even during relatively simple tasks (i.e., usual walking) (21, 26). The use of cognitive resources to compensate for PD related deficits is similar to previously reported theories of PD walking, which hypothesized that to compensate for movement automaticity deficits people with PD increased cognitive control (63), particularly executive control (26). However, executive-attentional deficits are common in PD (64) and these impact the ability to effectively compensate for underlying deficits. Therefore, when tasks become more challenging (such as dual-tasks, obstacles etc.) people with PD may not be able to effectively respond (24), which impacts gait and mobility, with implications for falls risk. Future studies may uncover further age or PD-related deficits with the use of more complex tasks that provide additional cognitive burden to participants.

Interestingly, although older adults had slightly greater PFC activation levels than young adults during walking, our findings were not significantly different which is in agreement to another previous fNIRS study (32). This demonstrated that healthy young and older adults may use their executive-attentional resources in a similar manner when walking, with little need to activate the PFC due to intact lower-level neural structures and activity compared to people with PD. Similarly, there were limited group differences found during the continuous 360° turning-in-place task, which may indicate that this complex task requires cognitive resources regardless of age or disease. Indeed, average PFC activation was higher in young adults, older adults and PD during continuous turning compared to walking, with PD participants having the highest HbO_2_ concentrations across groups. Interestingly, although there were no significant group differences, there were large between group effects for PFC activation during the early period of the turning task. This highlighted that young adults had much lower PFC activation during the early period of continuous turning than older adults or people with PD. Aging and PD may therefore lead to greater cognitive control being required to begin complex continuous motor tasks, however less cognitive control is required once the task underway.

### Clinical Interpretation

PFC activation appears to increase with walking in PD, but not with age. This may represent cortical compensation for sub-cortical dysfunction with PD. Findings suggest that targeting cortical activation, particularly executive-attentional activity within the PFC, with interventions such as pharmaceuticals, cueing strategies (visual, auditory or proprioceptive) or transcranial magnetic or direct current stimulation may help to alleviate the cortical burden of walking in PD. However, future studies are needed to establish findings and examine response to such interventions. Specifically, future studies should assess whether the observed increase in PFC activation is related to PD itself or to freezing of gait. In fact, as freezing of gait could represent a disruption of gait automaticity, people experiencing freezing of gait, even without PD, may show an increased cortical control of gait.

### Study Strengths and Limitations

A key strength of this study was the use of short-separation reference channels (1.5 cm apart) to account for the peripheral hemodynamic response that is associated with physical activity (44). Short-separation channels have only been used in one other mobile fNIRS study (65), but they should be used within future work to reduce noise and ensure repeatable findings. Another strength is that we have provided detailed data analysis steps from our fNIRS signal processing, whereas other previous studies often use ‘black-box' data analysis tools such as NIRS-SPM (66) or Homer2 (67).

A limitation of this study was that, despite being the first to asses test re-test reliability of fNIRS HbO_2_ measurement during walking and turning, test re-test analysis was only conducted in young adults. Reliability could possibly change in older adults or PD, as previous studies have shown this in patients with TBI (68). However, we may expect that reliability would even be higher in older adults and further in PD as, unlike young adults, they require more cognitive control of movement due to a loss of motor automaticity (1, 28), therefore findings in these groups may be more consistent. However, this is mainly speculative at this point, future studies could examine re-test reliability in older adult and PD using the same protocol developed here. Additionally, we did not examine the influence of disease stage, duration or other clinical factors (e.g., freezing of gait) in PD, which future studies should examine to provide greater understanding of cortical activation during walking and turning in PD.

## Conclusions

This study has demonstrated that the measurement of PFC activation during continuous turning and walking using a mobile fNIRS system with short-separation reference channels is repeatable. PFC activation during continuous turning and walking differs between young adults, older adults and PD, with greater activation required in PD compared to control groups for these motor tasks. Using the robust method developed in this study, future work could establish these findings within larger cohorts and examine the impact of more complex tasks on PFC activation.

## Ethics Statement

This study was carried out in accordance with the recommendations of Oregon Health & Science University IRB Committee with written informed consent from all subjects. All subjects gave written informed consent in accordance with the Declaration of Helsinki. The protocol was approved by the OHSU IRB.

## Author Contributions

SS and MM conceptualized the question and hypothesis. MM designed the study from which the data originates. SS, VB, and MM contributed to data collection and analysis. SS, VB, JQ, and MM contributed to the interpretation, writing and editing of the manuscript. SS wrote the first draft.

### Conflict of Interest Statement

The authors declare that the research was conducted in the absence of any commercial or financial relationships that could be construed as a potential conflict of interest.
